# The Effect of Granisetron on Sensory Detection and Pain Thresholds in Facial Skin of Healthy Young Males

**DOI:** 10.3389/fneur.2020.00237

**Published:** 2020-04-09

**Authors:** Malin Ernberg, Anna Wieslander Fältmars, Milad Hajizadeh Kopayeh, Sofia Arzt Wallén, Therese Cankalp, Nikolaos Christidis

**Affiliations:** Division of Oral Diagnostics and Rehabilitation, Department of Dental Medicine, Karolinska Institutet, and Scandinavian Center for Orofacial Neuroscience (SCON), Huddinge, Sweden

**Keywords:** granisetron, sodium channels, lidocaine, human, mechanical thresholds, thermal thresholds

## Abstract

**Background:** The specific serotonin type 3 (5-HT_3_)-receptor antagonist granisetron effectively reduces clinical as well as experimental muscle pain and hyperalgesia and with a duration that exceeds that of lidocaine. Hence, it may be an alternative to lidocaine as a local anesthetic. There are also some indications that granisetron in addition to 5-HT_3_ receptors blocks sodium channels. Thus, the local anesthetic effect by granisetron may resemble that of lidocaine, but this has not been tested. The aim of this study was therefore to compare the effect granisetron has on facial skin sensitivity to the effect of lidocaine and isotonic saline.

**Methods:** This was a randomized, controlled, and double-blind study, in which 1 ml of either granisetron (test-substance), lidocaine (positive control), or isotonic saline (negative control) was injected into the skin over the masseter muscle at three different occasions in 18 healthy males (27.2 ± 5.8 years old). Skin detection thresholds and pain thresholds for thermal stimuli as well as mechanical detection thresholds and sensitivity to a painful mechanical (pinprick) stimulus were assessed before (baseline) and 5, 20, 40, and 60 min after injection. The quality and area of subjective sensory change over the cheek were assessed 20 min after injection.

**Results:** All substances increased the mechanical detection threshold (granisetron: *p* = 0.011; lidocaine: *p* = 0.016; saline: *p* = 0.031). Both granisetron and lidocaine, but not isotonic saline, increased the heat detection thresholds (*p* < 0.001 and *p* < 0.02, respectively), but not the cold detection thresholds. Granisetron and lidocaine also reduced pinprick pain (*p* = 0.001 for each comparison). There were no significant differences between granisetron and lidocaine for any of these variables. There was no effect on thermal pain thresholds for any substance.

**Conclusion:** The similar analgesic patterns on mechanical sensory and pain thresholds as well as thermal sensory thresholds over the facial skin by subcutaneous injection of granisetron and lidocaine shown in this study and the absence of paresthesia, in combination with the reduced pain intensity and pressure pain sensitivity shown in previous studies, indicate that granisetron might be a novel candidate as a local anesthetic.

## Introduction

Serotonin (5-HT) is an important neurotransmitter involved in many diverse functions in the body, for example, sleep and awakening, appetite, aggression, and pain. 5-HT exerts its effect by activating different receptors, grouped into 5-HT_1_- to 5-HT_7_-receptors (1). The 5-HT_3_-receptor is widely distributed on neurons both in the brain and peripherally and participates in nociception supraspinally, spinally, and in the periphery (2–4).

5-HT_3_-antagonists, such as granisetron, ondansetron, and tropisetron, are commonly used treat radiotherapy- and chemotherapy-induced vomiting and nausea (5). In addition to this effect, several animal and human studies have shown that 5-HT_3_-antagonists reduce inflammatory and clinical pain, e.g., in fibromyalgia (6–9). In chronic orofacial myalgia, tender-point injections of granisetron were reported to be a safe and effective treatment (10). Further, systemic and local administration of granisetron increase the mechanical pain threshold over healthy human muscles (11–13).

It has been shown that granisetron has a higher affinity to the 5-HT_3_-receptor than ondansetron and tropisetron (1). However, there are also indications that granisetron and other 5-HT_3_-antagonists block sodium channels (14–18). Sodium channels play a key role in the activation of peripheral nociceptive sensory neurons involved in transmission of noxious stimuli (19, 20).

Local anesthetics, such as lidocaine, non-specifically block sodium channels, e.g., on nociceptors, mainly by inhibiting the sodium influx into the neuronal cell membrane, thus suppressing the cell excitability (21). Sodium channels on all types of sensory neurons are affected, but Aδ-fibers are most sensitive, followed by Aβ-fibers, and C-fibers (22, 23). This suppression leads to a transient loss of sensation in a circumscribed area of the body (24). In the orofacial region, local anesthetics are associated with paresthesia that lasts far beyond the duration of anesthesia (25). This paresthesia involves not just a perception of facial distortion (26) but also sensations of numbness, swelling, tingling, and itching (24). Based on this, a substance with local anesthetic effects, but without paresthesia would be desirable, especially for the orofacial region. Indeed, a previous *in vitro* and *in vivo* study showed that ondansetron blocked sodium channels in rat brain neurons, produced local analgesia in a dose-related manner, and caused numbness under the skin (16). Hence, 5-HT_3_ antagonists may perhaps be used as a new class of local anesthetics.

One way to investigate if granisetron produce local anesthetic-like effects in humans could be to record changes in skin sensitivity to mechanical stimuli (Aβ-fibers), heat stimuli (C-fibers), and cold stimuli (Aδ-fibers) after injection of the substance (16, 27–32). Hence, this study aimed to investigate the effect by granisetron on facial skin-sensitivity and comparing it to the effect of lidocaine and isotonic saline. We hypothesized that granisetron has a local-anesthetic like reduction of detection- and pain thresholds for mechanical and thermal stimuli.

## Materials and Methods

The present study was conducted at the Department of Dental Medicine, Karolinska Institutet, Huddinge, Sweden, between March 2014 and November 2018. It followed the present guidelines according to the Declaration of Helsinki and was approved by the Regional Ethical Review Board in Stockholm, Sweden (Dnr 2013/932-31/4) and the Swedish Medical Products Agency (EudoraCT-number 2008-000746-32). Verbal and written information of the study was given to all participants, and their written consent was obtained before the study start.

### Participants

The study is composed of 18 healthy male participants with a mean (SD) age of 27.2 (5.8) years. They were recruited by flyers posted at the Department of Dental Medicine, Karolinska Institutet, and at the library of Södertörn University, both in Huddinge, Sweden. According to the power calculation, based on the outcome from a previous study (10), inclusion of 17 participants would be sufficient to detect a difference of 30% (SD 30%) between interventions in order to reach a significance level (α) of 0.05 and a power (β) of 80%. In order to compensate for dropouts, one additional person was included.

Inclusion criteria were as follows: (a) age between 18 and 40 years, (b) good general health, and (c) male sex. Exclusion criteria were as follows: (1) any current pain from the orofacial region; (2) a diagnosis of temporomandibular disorders (TMD) according to the Diagnostic Criteria for TMD (DC/TMD) (33); (3) any type of headache; (4) diagnosed systemic muscular or joint diseases, such as fibromyalgia and rheumatoid arthritis; (5) whiplash-associated disorders; (6) neuropathic pain or neurological disorders; (7) pregnancy or lactation; (8) severe psychiatric conditions, including depression; (9) use of any kind of medication except for contraceptives 48 h preceding the study day; (10) use of any kind of medications, balms, or lotions affecting skin sensitivity; and (11) previous negative or allergic reactions to either of the injected substances (e.g., lidocaine or granisetron).

### Experimental Protocol

The study used a randomized, controlled, and double-blind design with each participant as his own control. The study consisted of three separate sessions, with at least 1 week of washout between. The sessions, in which granisetron (test-substance), lidocaine (positive control), and isotonic saline (negative control) were subcutaneously injected into the skin over the masseter muscle, were performed in a random order. The injections were placed subcutaneously in order just to affect the skin sensitivity and not the surrounding tissues. To randomize the order of injection, a randomization list was generated using a web-based randomization tool (www.randomization.com) by one of the researchers not participating in data collection (NC). With this randomization tool, not just the order of the sessions but also the side for injections were randomized in a balanced order. The injections were administered on the same side in each participant for all three substances.

The test substances were prepared in syringes prior to the experiment by the same researcher (NC). The syringes all appeared identical since all three substances were clear liquids, therefore making it impossible for the researchers collecting (SAW, TC) or registering (AW, MHK) the data, as well as for the participants to distinguish one substance from another.

**Figure 1:**
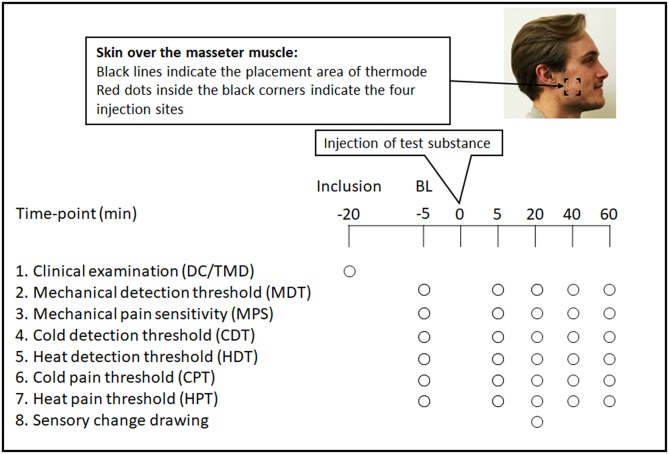
Flowchart showing the experimental protocol, and showing the time points in minutes and the order for every registration: inclusion examination, baseline (BL) and experimental assessments of mechanical detection threshold (MDT), mechanical pain sensitivity (MPS), cold detection threshold (CDT), heat detection threshold (HDT), cold pain threshold (CPT), heat pain threshold (HPT), and drawing of sensory change as well, in 18 healthy males. Written consent to publish image was obtained.

The participants sat in a conventional dental chair in a quiet environment during the entire experiment. Before the experiment started, they underwent a clinical examination according to the DC/TMD in order to screen for trial suitability and inclusion (33). Subsequently, after inclusion as well as in the beginning of each session, baseline recordings of mechanical detection threshold (MDT), mechanical pain sensitivity (MPS) to a pinprick stimulus, and detection as well as pain thresholds to cold (CDT and CPT, respectively) and heat (HDT and HPT, respectively) were recorded in the same order for all participants and sessions. The participant had his eyes closed during the recordings and was asked to concentrate on the task. After the baseline registrations, an injection of the test substance followed. The recordings were then repeated in the same order (MDT, MPS, CDT, HDT, CPT, HPT) after 5, 20, 40, and 60 min. Twenty minutes after the injection, the participants were also asked to make a drawing of the area of subjective sensory change over the cheek at the site of injection. The experimental protocol is shown in Figure 1.

### Injections

A felt pen was used to mark out the area of injection overlying the masseter muscle using a plastic patch cut to a size equivalent to the size of the thermal probe (3 × 3 cm), which was used for assessment of thermal sensitivity. The plastic patch was placed in the midline of the masseter muscle and 1 cm above the lower border of the mandible. This location was used for all sessions in order to ensure that the injections were placed at the same sites all three times. The skin overlying the masseter muscle was chosen since it is an easily accessible orofacial area that has been used in previous experiments by our research group.

The substances used in the present study were as follows: (a) the active test substance granisetron (Kytril® 1 mg/ml, Roche, Stockholm, Sweden, pH ranging from 4.0 to 6.0); (b) the positive control substance (for the possible analgesic effect) was lidocaine (Xylocain® 20 mg/ml, AstraZeneca AB, Södertälje, Sweden, pH ranging from 5.0 to 7.0); and (c) the negative control substance isotonic saline (NaCl 9 mg/ml B Braun, Melsungen, Germany, pH ranging from 4.5 to 7.0). Isotonic saline was used as vehicle for both granisetron and lidocaine. One milliliter of the chosen substance was subcutaneously injected using a 19-mm needle (Neofly, BOC Ohmeda AB) with a 0.4-mm diameter from a 2-ml syringe. The solution was injected into four different sites, i.e., 0.25 ml in each site, 3 mm inside each corner of the marked area using the above-described plastic patch. This was done in order to allow the injected substance to diffuse into the entire area that was covered by the thermal probe. The spread of the substances caused an ischemic appearance and was homogeneous. One single bolus did not diffuse more than 1 × 1 cm, thus not covering the entire surface of the thermal probe. All substances had room temperature, i.e., 23°C.

### Assessment of Mechanical Detection and Pain Level

The MDT was assessed using calibrated von Frey nylon monofilaments (Anesthesiometer, Somedic Sales AB, Hörby, Sweden) exerting bending forces ranging from 0.026 to 110 g according to the stepwise ascending–descending method in order to find the lowest detectable bending force. This means that the examiner started with the lightest monofilament by placing it perpendicular to the skin surface with a contact time of 1.5 s. If no sensation was reported, the examiner continued to apply filaments in ascending order in the same manner until the participant first reported a sensation. The weight of this filament was noted. The whole procedure was repeated twice and the mean value of the monofilament weight for the three assessments was calculated and used for statistical analysis. In order to assess MPS, the von Frey nylon monofilament 19 with a force of 110 g was used. The filament was applied three times during 1.5 s. Directly after each application, the intensity of the pinprick stimulus applied was assessed on a 0–100 numerical rating scale (NRS), where 0 means no sensation, 50 is just barely painful (pain threshold), and 100 represents the most painful sensation one can imagine (34). The mean value of the three ratings was used for further analysis.

### Assessment of Thermal Detection and Pain Threshold

In order to assess the thermal detection as well as pain thresholds, an electronic thermo-test system was used (CHEPS thermo-test system, Medoc Ltd. Ramat Yishai, Israel). The measurements were done using an advanced thermal stimulator (ATS) with a contact area of 3 × 3 cm that was placed on the skin surface of the masseter. A preset automatic program was used in which the thermal stimulator had a baseline temperature of 32°C (skin temperature) and a minimum and maximum temperature of 0° and 55°C, in order not to cause frostbite or burn of the skin. The automatic program started with recording of the cold detection threshold (CDT), i.e., cooling of the skin. The participant was asked to press a stop button as soon as he experienced that the thermode started to get cold. The temperature then increased to 32°C to start a new cycle. This was done four times with an interstimulus interval (ISI) of 4 s and a decrease/increase rate of 1°C/s. The warmth detection threshold (WDT) was then immediately assessed in the same manner. Directly after assessments of thermal detection thresholds, the cold pain threshold (CPT) and heat pain threshold (HPT) were recorded, i.e., the cooling/heating continued until the cold or heat became minimally painful. The pain thresholds were assessed three times, with an ISI of 10 s, and a decrease/increase rate of 1.5°C/s. The average of the repeated recordings was used in the analyses.

### Assessment of Sensory Change Drawings

Twenty minutes after the injections, each participant was asked to mark out the maximum perceived area of subjective change in sensory experience over the injected cheek. The participants were instructed to encircle the area(s) where they perceived any kind of sensory change with a felt pen on a printed paper sheet with two images, one image displaying a lateral view of the head for the side of injection and one image displaying an intra-oral lateral view of the head highlighting the teeth and jaws for the side of injection (Figures 1, 3). Finally, the participants were asked to describe any type of sensory change.

For the analysis of the subjective sensory change drawings, each picture was scanned separately using a network printer (Ricoh MP C6004ex) having a resolution of 300 dpi. The area of subjective change in sensory experience was then calculated with an area calculation function in a photo editing program (Adobe Photoshop CS4 Extended version 11.0.2, Adobe Systems Incorporated USA). The areas of the sensory change drawings are expressed in arbitrary units (au).

### Data Analysis and Statistics

Data were analyzed using the SigmaPlot for Windows version 14.0 software (Systat Software Inc., San Jose, CA, USA). The normality of the data was tested using the Shapiro–Wilk's test. Parametric statistical methods were used for normally distributed data on a continuous scale, while non-parametric statistics were used for data that were not normally distributed or on an ordinal scale. All pain sensitivity variables (MPS, CPT, and HPT) were normally distributed, so parametric statistical methods were used to analyze these data. All detection thresholds (MDT, CDT, and HDT) were not normally distributed. An attempt to log transform the data did not result in normal distribution of data; this is why non-parametric methods were used. Means and standard deviations (SD) were used for descriptive statistics for pain variables, whereas medians and interquartile ranges (IQRs) were used for detection thresholds. The level of significance was set at *p* < 0.05.

Two-way ANOVA for repeated measures (RM ANOVA) with time and substance as factors analyzed group differences in MPS, CPT, and HPT. When a significant difference was indicated, Tukey test for multiple comparisons vs. a control group (baseline) was used as *post hoc* test to test for differences between substances and time points. Changes of CDT, HDT, and MDT across times were analyzed using Friedman ANOVA with Dunn's test for multiple comparison vs. a control group (baseline) as *post hoc* test. Friedman test was also used to analyze differences between the substances at the different time points. As there were in total five comparisons made for these variables, Bonferroni correction was done to compensate for multiple testing, giving a significance level of *p* < 0.01 in these analyses.

## Results

All 18 participants completed all three sessions, i.e., there were no dropouts.

There were no significant differences between the baseline values (means and medians) of MDT, MPS, CDT, HDT, CPT, or HPT across the three sessions, i.e., before injection of either granisetron, lidocaine, or isotonic saline (Table 1).

**Table 1:** Baseline values in mean (SD) and median (IQR) of cold detection threshold (CDT), heat detection threshold (HDT), and mechanical detection threshold (MDT), as well as cold pain threshold (CPT), heat pain threshold (HPT), and mechanical pain level (MPS) are presented.

	**Isotonic saline**	**Granisetron**	**Lidocaine**
**DETECTION THRESHOLDS**			
Mechanical detection (MDT) (g)			
Mean (SD)	4.9 (3.1)	6.4 (10.7)	5.1 (3.7)
Median (IQR)	3.4 (3.8)	3.4 (3.8)	3.4 (3.8)
Cold detection (CDT) (°C)			
Mean (SD)	30.2 (1.3)	30.0 (1.5)	31.6 (1.9)
Median (IQR)	30.5 (1.0)	30.3 (1.4)	31.0 (3.0)
Heat detection (HDT) (°C)			
Mean (SD)	34.6 (1.6)	34.0 (1.2)	34.4 (2.0)
Median (IQR)	33.8 (2.4)	33.5 (1.1)	33.6 (1.5)
**PAIN THRESHOLDS**			
Mechanical pain sensitivity (MPS) in NRS 0–100			
Mean (SD)	36.1 (14.6)	39.2 (16.3)	38.6 (14.5)
Median (IQR)	37.5 (30.0)	37.5 (24.5)	40.0 (21.4)
Cold pain (CPT) (°C)			
Mean (SD)	13.6 (11.0)	14.1 (9.6)	12.3 (9.8)
Median (IQR)	15.4 (22.6)	15.6 (20.9)	11.3 (19.8)
Heat pain (HPT) (°C)			
Mean (SD)	44.2 (4.6)	44.0 (4.4)	45.4 (4.0)
Median (IQR)	45.2 (7.9)	43.3 (6.9)	46.1 (6.2)

*SD, standard deviation; IQR, interquartile range (75th percentile minus 25th percentile); °C, degrees in Celsius; g, gram; NRS, numeric rating scale. There were no significant differences in any aspect*.

### Mechanical Detection Threshold

**Figure 2:**
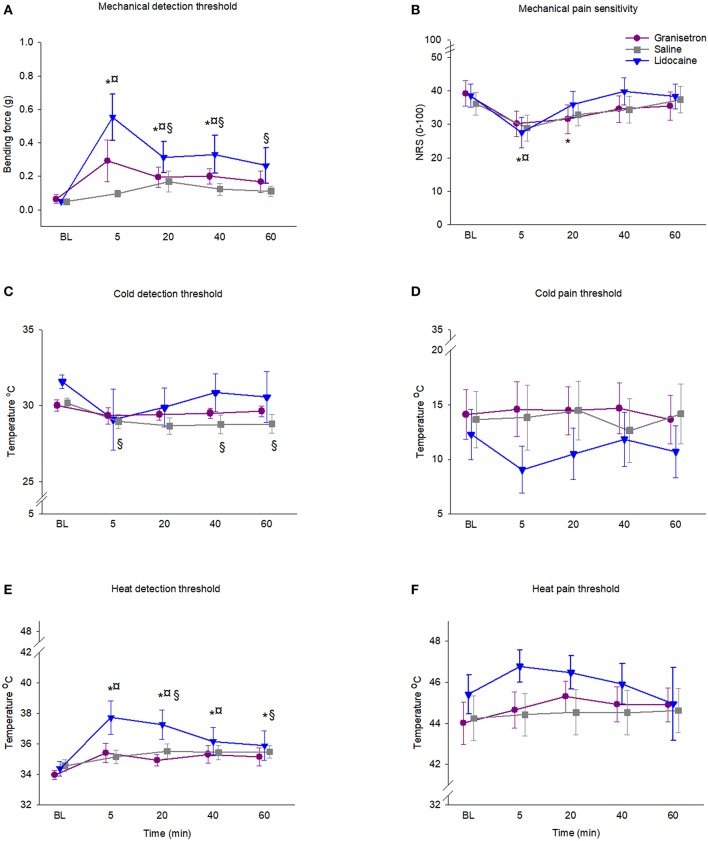
Graph showing the changes in skin sensitivity after subcutaneous injection of granisetron (test substance), lidocaine (positive control), or saline (negative control) into the skin overlying the masseter muscle in 18 healthy males. **(A)** Mechanical detection threshold (MDT), **(B)** mechanical pain sensitivity (MPS), **(C)** heat detection threshold (HDT), **(D)** heat pain threshold (HPT), **(E)** cold detection threshold (CDT), and **(F)** cold pain threshold (CPT). Mean (SEM) values are presented. In order to clarify, the time points for each substance are differentiated. The special characters indicate significant differences compared to baseline (*p* < 0.05) for *granisetron, ^**¤**^lidocaine, and ^§^saline.

Significant changes in MDT were recorded over time for all substances, as shown in Figure 2A.

MDT for granisetron increased with time (*p* < 0.001; Friedman ANOVA). The *post hoc* test showed that the increase was significant compared to baseline at all time points during 40 min after injection (*p* < 0.011; Dunn's test). The increase of MDT was 458% at 5 min after injection, 304% at 20 min after injection, and 313% at 40 min after injection. The lidocaine injection increased the MDT (*p* < 0.001; Friedman ANOVA). The increase of MDT after injection with lidocaine followed the same pattern as granisetron and was significant during 40 min (*p* < 0.016; Dunn's test) and was 1088% at 5 min after injection, 616% at 20 min, and 652% after 40 min. Also, isotonic saline injection increased the MDT (*p* < 0.001; Friedman ANOVA). The increase was significant from 20 min up to 60 min after injection (*p* < 0.031; Dunn's test) and was 347% at 20 min after injection, 253% at 40 min, and 228% at 60 min after injection.

There were no significant differences in MDT between the substances at the different time points (*p* > 0.01; Friedman ANOVA).

### Mechanical Pain Sensitivity

Lidocaine and granisetron, but not isotonic saline, reduced the MPS. The two-way RM ANOVA showed a time effect (*df* = 4; *F* = 23.03; *p* < 0.001), but no difference between substances (*df* = 2; *F* = 2.95; *p* = 0.066). However, there was an interaction between time and substance (*df* = 8; *F* = 5.06, *p* < 0.001). The *post hoc* test showed that granisetron reduced the MPS 5 and 20 min after injection by 23 and 20%, respectively (*p* = 0.001, Tukey test) compared to baseline, while lidocaine reduced the MPS 5 min after injection (*p* = 0.001, Tukey test). No significant changes were found over time for isotonic saline (Figure 2B).

There were no significant differences in changes of MPS between the substances at the different time points (*p* > 0.05; Tukey test).

### Cold Detection Thresholds

There were no significant differences in CDT over time for granisetron (*p* = 0.065; Friedman ANOVA) or lidocaine (*p* = 0.839; Friedman ANOVA), as shown in Figure 2C. A significant decrease of CDT was recorded for isotonic saline when compared to baseline (*p* < 0.001; Friedman ANOVA). According to the *post hoc* test, the decrease was significant at 5, 40, and 60 min after injection (*p* < 0.005; Dunn's test). The decrease was 4% at 5 min after injection, 5% at 40 min, and 5% at 60 min after injection.

There were no significant differences in changes of CDT between the substances at the different time points (*p* > 0.059; Friedman ANOVA).

### Cold Pain Threshold

Three of the participants reached the minimum preset temperature without reporting any pain; i.e., they did not reach the CPT. Their data were therefore regarded as missing data, so the CPT results are based on 15 participants. None of the injected substances affected the CPT. The two-way RM ANOVA did not show any significant time effect (*df* = 4; *F* = 0.28; *p* = 0.888) or any difference between substances (*df* = 2; *F* = 2.04; *p* = 0.146). Neither was there any interaction between time and substance (*df* = 8; *F* = 1.26, *p* = 0.269) (Figure 2D).

### Heat Detection Threshold

A significant increase in HDT was recorded over time for all substances, as shown in Figure 2E. HDT for granisetron increased significantly when compared to baseline (*p* < 0.001; Friedman ANOVA). The *post hoc* test showed that the increase was significant at all time points after injection (*p* < 0.001; Friedman ANOVA) and was 4.0% at 5 min after injection, 2.8% at 20 min after injection, 4.0% at 40 min after injection, and 3.5% at 60 min after injection. The lidocaine injection significantly increased the HDT when compared to baseline (*p* < 0.001; Friedman ANOVA). The *post hoc* test showed that the increase was only significant at 40 min after injection (*p* < 0.001; Friedman ANOVA), and was 5.2%. There was a significant increase in HDT after injection of isotonic saline, when compared to baseline (*p* < 0.011; Friedman ANOVA). The *post hoc* test showed that the increase was significant at 20 and 60 min after injection (*p* < 0.031; Friedman ANOVA), and was 2.7% after 20 min and 2.6% after 60 min.

There were no significant differences in the magnitude of the changes of HDT between the substances (*p* > 0.01; Friedman ANOVA).

### Heat Pain Threshold

None of the injected substances affected the HPT. The two-way RM ANOVA did not show any significant time effect (*df* = 4; *F* = 2.10; *p* = 0.090) or any difference between substances (*df* = 2; *F* = 1.80; *p* = 0.180). Likewise, there was no interaction between time and substance (*df* = 8; *F* = 1.18, *p* = 0.318), as shown in Figure 2F.

### Area of Sensory Changes

**Figure 3:**
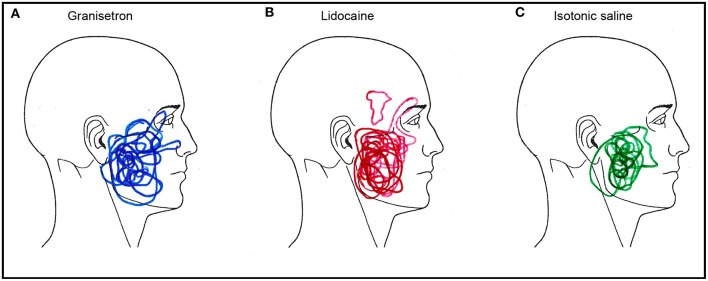
Superimposed illustrations of the areas of the changes in sensory experience after subcutaneous injections of **(A)** granisetron (blue; active test substance), **(B)** lidocaine (red; positive control substance), and **(C)** isotonic saline (green; negative control substance) in 18 healthy males. Note that 16 male participants marked out their change in sensory experience after injection of granisetron, while all 18 did so after injection of lidocaine and 14 did so after injection with isotonic saline. These are not missing data, but a sign of no change in sensory experience.

All injected test substances induced an experience of sensory change over the skin surface. The sensory experience was described by the participants as (a) numbness (*n* = 15) and tingling (*n* = 10) for granisetron; (b) numbness (*n* = 18), swelling (*n* = 18), tingling (*n* = 16), and itching (*n* = 5) for lidocaine; and (c) numbness (*n* = 7) and tingling (*n* = 8) for isotonic saline. Several of the participants experienced more than one sensory change. The area of subjective change in sensory experience over the skin overlaying the injections was 107.4 au after injection of lidocaine, 73.8 au after injection of granisetron, and 62.8 au after the isotonic saline injection. The perceived affected area 20 min after lidocaine injection was 159% larger than after isotonic saline (*p* = 0.039; Friedman ANOVA) and 91% larger than after granisetron (NS). The perceived affected area 20 min after granisetron was 35% larger than after isotonic saline (NS). Superimposed illustrations of the areas of the sensory changes are presented in Figure 3.

## Discussion

In this study, the effect on skin sensitivity by a subcutaneous injection of granisetron was compared to lidocaine and isotonic saline. The main finding was that the effect by granisetron on facial skin sensitivity resembles that of lidocaine. Both substances decreased the sensitivity to mechanical stimuli and increased the sensitivity to heat. The changes in sensitivity were greater for lidocaine, but granisetron showed a somewhat longer duration. Thus, granisetron could therefore be considered a novel candidate for local anesthetics, without the bothersome side effect of traditional local anesthetics, such as lidocaine, in terms of facial distortion, i.e., swelling lasting beyond the duration of the anesthesia.

All substances significantly increased the MDT and HDT, while only lidocaine and granisetron increased the MPS. All substances also induced an area of perceived sensory change over the skin surface with a significant difference between lidocaine and isotonic saline, but not between granisetron and saline or lidocaine. The increase for all outcomes was the greatest for lidocaine and more pronounced for mechanical stimuli than for heat. The increase in MDT for granisetron showed a similar pattern to lidocaine. The effect of lidocaine was expected based on results from numerous previous studies showing a local anesthetic effect by lidocaine (35–37). The increase in HDT regarding lidocaine is further supported by another study using a similar methodology to ours (30) and by recent studies using quantitative sensory testing in the facial area of healthy participants after topical lidocaine application (38, 39). However, although a pain-reducing effect by granisetron has been reported in many previous human studies (10, 12, 40), the finding that granisetron showed a similar effect to lidocaine on skin sensitivity, even if it was less pronounced, is a new and interesting finding. This differs from findings in 5-HT_3_ knock-out mice, where neither thermal, nor mechanical nociceptive thresholds were affected during physiologic conditions (41). An explanation to the difference could be additional blocking of other receptors and ion channels by granisetron (14–17). The fact that also isotonic saline showed weak effects on skin sensitivity could perhaps be explained by a change of sodium and chloride ions in the tissue that was sufficient to minorly change nerve conduction.

Another interesting finding was that the duration of the reduced skin sensitivity was longer for granisetron than for lidocaine, regarding MDT, MPS, and HDT. For example, the decrease in MPS by lidocaine peaked at 5 min after injection and thereafter rapidly declined and was non-significant already after 20 min. In comparison, after injection of granisetron, the decrease in MPS lasted beyond 20 min. Similar results, with a longer pain-reducing effect by granisetron than lidocaine, have been reported in patients with rheumatic diseases using tropisetron (40). The results are also in accordance with a previous study from our group where granisetron injected into the masseter muscle increased the pressure pain threshold of healthy volunteers (12). One possible explanation to this could be differences in elimination half-times between the substances where granisetron has a half time of ~9 h (42) and lidocaine only 90–110 min (43). Another possible explanation for the longer duration of granisetron than lidocaine can be the effect on 5-HT_3_-receptors, which reduces 5-HT-enhanced hyperalgesia to thermal stimuli (44). A third possible explanation is that calcium influx by granisetron, which causes longer-lasting phosphorylation of sodium channels promoting further activation (45).

Neither lidocaine nor granisetron significantly affected the CDT. We had expected an effect by lidocaine on CDT since this has been reported before (39). In that study, topical application of lidocaine reduced CDT with 8°C. However, in our study the changes were much smaller for all substances and both lidocaine and granisetron also showed minor changes with time, although not significant. Our findings are also consistent with findings by Krumova et al. where the change in CDT after lidocaine application, even if significant, were small (30). The reason for the diverging results regarding lidocaine could perhaps be methodological differences, such as selected dose and way of distribution. We used a single injection of lidocaine, while in the other studies, lidocaine cream was applied topically for 30 min and 6 h, respectively (30, 39). In contrast, isotonic saline reduced the CDT. This is an intriguing finding, since saline was used as negative control and thus not expected to evoke any changes in the variables. However, the changes of CDT were small for all substances, although the greatest for lidocaine. However, in comparison to isotonic saline, lidocaine showed much greater interindividual variation. Granisetron, on the other hand, showed a similar interindividual variation as isotonic saline and also a trend toward significance. This could therefore be a plausible explanation for the significant effect only for isotonic saline.

All substances had some effect on sensory thresholds and there were no significant differences between the substances at any time point. One explanation for this could be that the subcutaneous bolus of the injected substances increased the tissue pressure to such an extent that that the bolus itself affected nerve signaling (46, 47), which then disguised any difference between substances.

Based on the similarities in local anesthetic effect between granisetron and lidocaine, it is tempting to speculate about the mechanisms. Granisetron has a high affinity to the 5-HT_3_ receptor, but there are also indications that it blocks, e.g., voltage-gated sodium channels (14–17). Hence, the local anesthetic-like effect by granisetron could be due to a dual blockage of both 5-HT_3_-receptors and sodium channels. Lidocaine non-selectively blocks sodium channels, but it might be that granisetron in the periphery more selectively blocks sodium channels involved in pain transmission, i.e., Nav 1.8 and Nav 1.9. If so, this could hypothetically explain the lack of paresthesia. However, since the exact function of the different voltage-gated sodium channels is unknown, as well as the mechanisms behind paresthesia after lidocaine injection, these could be possible areas for future research. In addition, there are indications that 5-HT_3_ antagonists also block other ion channels, such as potassium, calcium, and acid-sensing ion channels (48, 49), so the local anesthetic effect may not be limited to 5-HT_3_ receptor and sodium channel blocking.

### Study Strengths and Limitations

Some study strengths and limitations will be addressed. Firstly, there were two researchers performing the examinations and injections (SAW and TC), which could be considered as a limitation. However, in order to minimize any risk of bias, both investigators were trained by the same researcher (NC) and used the same plastic patch during all sessions to ensure that the injections were placed at the same spot. The bolus of an injected substance could increase the pressure in the tissue and affect the ability to mediate nerve signals (46, 47). Any such effect on the outcome in this study can be ruled out due to the study design since the same volume of all substances was injected and the injections were made in the same site and with the same (subcutaneous) depth. The latter also rules out any potential risk of activation of centrally mediated descending pain inhibitory pathways caused by tissue damage from the needle insertion (50–52), because it requires tissue damage of muscle fascia and tissue (53). Secondly, the present study only included young healthy males, which also could be seen as a limitation since the results cannot be extrapolated to females. Therefore, future studies including both genders are necessary. A third and final limitation was an observed difficulty regarding the methodology. Some of the participants found it difficult to identify the exact transition from non-painful to painful thermal sensation, especially regarding CPT. The consequence was that three of the participants reached the preset minimum temperature (0°C) for all of the three assessments and for all substances without reaching the CPT, why their data could not be included in the statistical analyses. One could argue that this would underpower the study leading to a type II error; however, this was not the case since power was checked for and reached over 90%. On the other hand, there were no significant changes of HPT either, although data were based on all participants. In addition, our results are in line with those of Krumova et al. (30), in which thermal pain thresholds did not even change significantly after application of a lidocaine patch for 6 h in 26 healthy participants. Finally, it would probably not affect the outcome regarding differences between substances.

## Conclusion

In conclusion, the findings of this study suggest that a subcutaneous injection of granisetron into the facial skin decreases the sensory transmission, mainly concerning the painful mechanical and heat detection stimuli. The analgesic-like effects by granisetron lasted longer than for lidocaine and lacked the bothersome side effects in terms of numbness that outlasts the analgesic effect seen after lidocaine injection. Combined, these results strengthen the role of granisetron as a promising local anesthetic, especially for use in the orofacial area. However, further research with more subjects and with both sexes included is necessary for more profound conclusions.

## Data Availability Statement

The datasets generated for this study are available on request to the corresponding author.

## Ethics Statement

The studies involving human participants were reviewed and approved by Regional Ethical Review Board in Stockholm, Sweden (Dnr 2013/932-31/4) Swedish Medical Products Agency (EudoraCT-number 2008-000746-32). The patients/participants provided their written informed consent to participate in this study. Written informed consent was obtained from the participant for the publication of Figure 1.

## Author Contributions

ME designed the research, participated in data analysis, and drafted the manuscript. AW and MH collected the research data, participated in data analysis, and participated in results discussion and manuscript editing. SA and TC performed the research, participated in data analysis, and participated in results discussion and manuscript editing. NC designed the research, analyzed the data, participated in results discussion, and drafted the manuscript.

### Conflict of Interest

The authors declare that the research was conducted in the absence of any commercial or financial relationships that could be construed as a potential conflict of interest.
